# Modified Gray-Level Haralick Texture Features for Early Detection of Diabetes Mellitus and High Cholesterol with Iris Image

**DOI:** 10.1155/2022/5336373

**Published:** 2022-04-20

**Authors:** Rinci Kembang Hapsari, Miswanto Miswanto, Riries Rulaningtyas, Herry Suprajitno, Gan Hong Seng

**Affiliations:** ^1^Department of Informatics, Faculty of Electrical and Information Technology, Institut Teknologi Adhi Tama Surabaya, Indonesia; ^2^Department of Mathematics, Faculty of Sciences and Technology, Universitas Airlangga, Surabaya, Indonesia; ^3^Department of Physics, Faculty of Sciences and Technology, Universitas Airlangga, Surabaya, Indonesia; ^4^Department of Data Science, Universiti Malaysia Kelantan, 16100 UMK City Campus, Pengkalan Chepa, Kelantan, Malaysia

## Abstract

Iris has specific advantages, which can record all organ conditions, body construction, and psychological disorders. Traces related to the intensity or deviation of organs caused by the disease are recorded systematically and patterned on the iris and its surroundings. The pattern that appears on the iris can be recognized by using image processing techniques. Based on the pattern in the iris image, this paper aims to provide an alternative noninvasive method for the early detection of DM and HC. In this paper, we perform detection based on iris images for two diseases, DM and HC simultaneously, by developing the invariant Haralick feature on quantized images with 256, 128, 64, 32, and 16 gray levels. The feature extraction process does early detection based on iris images. Researchers and scientists have introduced many methods, one of which is the feature extraction of the gray-level co-occurrence matrix (GLCM). Early detection based on the iris is done using the volumetric GLCM development, namely, 3D-GLCM. Based on 3D-GLCM, which is formed at a distance of *d* = 1 and in the direction of 0°, 45°, 90°, 135°, 180°, 225°, 270°, and 315°, it is used to calculate Haralick features and develop Haralick features which are invariant to the number of quantization gray levels. The test results show that the invariant feature with a gray level of 256 has the best identification performance. In dataset I, the accuracy value is 97.92, precision is 96.88, and recall is 95.83, while in dataset II, the accuracy value is 95.83, precision is 89.69, and recall is 91.67. The identification of DM and HC trained on invariant features showed higher accuracy than the original features.

## 1. Introduction

An image can be used as a medium to store data and it can also store information. A visual image consists of an object that is not easy to be interpreted by the eye and interpreted by the brain. One way to recognize an image is to know the features inherent in an object. Several features are often used for feature extraction, including shape features, color features, and texture features. Image texture can be characterized by density, regularity, uniformity, frequency, directionality, and roughness [1].

The feature extraction process obtains characteristics or texture characteristics. Texture analysis plays an essential role in digital image processing [2, 3]. Computerized texture analysis is based on the statistical or structural properties of spatial patterns. The statistical approach considers that a two-dimensional random field generates the intensity. This technique is based on spatial frequencies and produces texture characteristics. Examples of methods include run length, autocorrelation, co-occurrence, and edge frequency. At the same time, structural techniques are related to the preparation of small parts (primitives) of an image. The structural analysis method describes the nature and placement of texture elements; an example of the technique is the fractal model.

Texture-based features are better than wavelet-based features by classifying in general. Features based on first- and second-order statistics characterizing textures were used for brain image classification. Both are significantly better than methods based on wavelet transform [4]. Ershad, S.F extracted textural and statistical information from the nucleus and cytoplasm on cell images. By combining the Haralick features, global significant values and time series features are used for feature extraction. This study is efficient for detecting cervical cancer using pap-smear images [5].

The medical use of image textures has been reported in the previous studies. Examples are early detection of Alzheimer's disease [6], dengue fever [7], osteoarthritis [8, 9], and lung cancer [10, 11], while research on disease diagnosis based on iris image feature extraction is used to diagnose kidney disease [12] and heart disease [13], detect Alzheimer's [14], detect stomach disorders [15], and others.

There are many texture information approaches. The approaches include the convolutional neural network, the local binary pattern, the wavelet, the multiscale patch-based recognition method, and the gray-level co-occurrence matrices (GLCM). Haralick texture features calculated from GLCM have been successfully applied in disease classification and detection. The examples are the detection of brain tumors [16]; lung diseases [17]; classifying kidney images such as normal, kidney stones, kidney cysts, and kidney tumors [18]; and COVID-19 detection [19].

2D texture information cannot represent the actual texture of 3D objects in 3D space. The textures reflect variations or repetitions of spectral values in the area, and the relative positions of primitives in the 2D image region cannot reflect genuine relationships in the 3D space. Therefore, the 3D texture will be more robust against variations in viewing angles. In medical image processing, 3D gray-level co-occurrence matrices (3D-GLCM) have been used to analyze renal cell carcinoma tissue [20], analyzing the state-of-the-art art in 3D biomedical texture analysis to identify specific needs [21], classification of benign and borderline favor types on follicular neoplasm images [22], identify the kind of ovarian tissue [23], classification of polyps by CT colonography [24], and identification of DM and HC based on iris image [25].

In this paper, we explore the potential of 3D-GLCM to identify patients with diabetes mellitus (DM) and high cholesterol (HC). It is an alternative to noninvasive detection of diabetic and/or high cholesterol patients. A comprehensive interpretation of 3D-GLCM-based texture features modified asymptotically invariant to image quantization in the context of DM and/or HC detection is presented.

The modification of Haralick's gray-level feature extraction in this study was applied to the image of the eye's iris. Furthermore, an evaluation of the original 3D-GLCM and invariant 3D-GLCM feature extraction was carried out. In addition, this feature extraction modification was also tested for other image objects, namely, MRI of brain tumors and X-ray of pneumonia, to determine the accuracy of the proposed method. The remainder of this paper is organized as follows.  Section 2 describes the proposed feature extraction materials and techniques; Section 3 describes the results of the method process; Section 4 discusses the findings obtained compared to previous related research. Finally, conclusions are drawn in Section 5.

## 2. Material and Methods

The workflow of the proposed model consists of three steps. The first step is to convert the RGB value of the iris image into the gray-level value of the entire dataset. The second step forms the 3D-GLCM image features from the converted iris image, the result of step one. Finally, in the third step, 3D-GLCM and 3D-GLCM Invariant models identify the iris image. Details of the three phases are presented below.

### 2.1. Grayscale Image Conversion

In computing, a grayscale digital image is an image in which the value of each pixel is a single sample. The displayed image of this type of image consists of a gray color, where it varies for black at the weakest intensity and white at the most vigorous intensity, but the color variations differ very much.

Grayscale images are often a calculation of the light intensity of each pixel in a single band electromagnetic spectrum.

**Figure 1:**
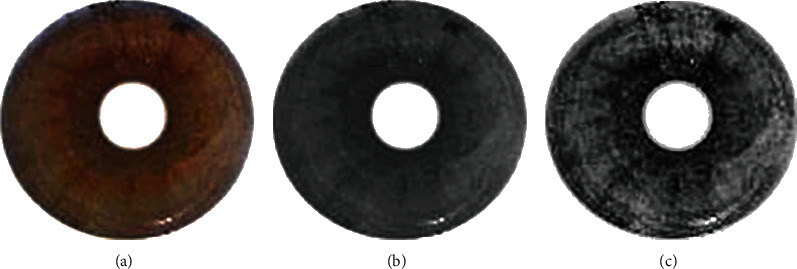
Iris images: (a) original RGB iris images, (b) grayscale conversion iris images, and (c) iris image enhancement with AHE.

In this study, the data source is an RGB image of type .jpg. The image is converted to the function contained in Matlab2019a, namely, the rgb2gray() function. The gray-level scaling is a trade-off between reducing the disparity and retaining sufficient information to identify the iris. Therefore, the gray-level scaling is essential to improve identification performance. This study used the adaptive histogram equalization (AHE) method [26–28] for image improvement.  Figure 1 shows the results of the conversion process of RGB iris images to grayscale iris images and the enhancement of iris images using the AHE method.

### 2.2. 3D Gray-Level Co-occurrence Matrix (3D-GLCM)

The gray-level co-occurrence matrix (GLCM), commonly known as 2D-GLCM, was first proposed by Haralick with 28 features to explain spatial patterns [29]. GLCM can reflect comprehensive information from the image area by computing the correlation between the intensity of two pixels, namely, reference and neighboring pixels, with a certain distance and direction.

GLCM uses texture calculations in the second order. Texture measurements in the first order using statistical analyses are based solely on the pixel values of the original image, such as variance. They ignore the pixel adjacency relationship. In the second order, the relationship between pairs of two actual image pixels is taken into account, where it pays attention to the relationship between the reference pixel and neighboring pixels.

(1) $$\begin{array}{c} P_{COM}\left(i,j,k,d,\theta \right)=N\langle \left(\left(x_{n1},y_{n1}\right),\left(x_{n2},y_{n2}\right),\left(x_{n3},y_{n3}\right)\right)\in M_{1}xM_{2}xM_{3}\left|\begin{array}{c} \max\left(\left|x_{n1}-x_{n2}\right|,\left|y_{n1}-y_{n2}\right|\right)=d\\ \max\left(\left|x_{n2}-x_{n3}\right|,\left|y_{n2}-y_{n3}\right|\right)=d\\ \Theta \left(\left(x_{n1},y_{n1}\right),\left(x_{n3},y_{n3}\right)\right)=\theta \\ I\left(x_{n1},y_{n1}\right)=i\\ I\left(x_{n2},y_{n2}\right)=j\\ I\left(x_{n3},y_{n3}\right)=k \end{array}\right.\rangle . \end{array}$$

GLCM is a matrix with the size *N* × *N*, where *N* is the level of gray intensity in the image. The sum of the intensity of the pixel pairs with a certain distance and direction becomes the matrix element. Given that *G*_*p*_(*i*, *j*, *k*, *θ*) represents the 2D-GLCM of pixel *p* with a distance and direction *θ*, (*i*, *j*) is the number of rows and columns in the matrix.

The extension of 2D-GLCM to volumetric images has been proposed in [30, 31], known as 3D-GLCM. The 3D-GLCM is similar to the 2D-GLCM variant, but the displacement vector is defined using three *x*, *y*, *z* coordinates and a higher number of orientations. The relative distance is determined by vector **d** = [*d*_1_, *d*_2_, *d*_3_], where the vector points from the reference pixel to the neighboring pixel. It can be described in equation (1) [32].

*P*_*COM*_ is the 3D-GLCM matrix element; *i* is the reference pixel value; *j* is the first neighbor pixel value; *k* is the second neighbor pixel value; *d* is the distance between pixels, is the direction formed by the reference pixel, and the second neighbor pixel; *N* is the frequency of occurrence of the pair of pixels; (*x*_*n*1_, *y*_*n*1_) are the coordinates of the reference pixels; (*x*_*n*2_, *y*_*n*2_) are the coordinates of the first neighboring pixel; and (*x*_*n*3_, *y*_*n*3_) are the coordinates of the second neighboring pixels. *M*_1_, *M*_2_, and *M*_3_ are the number of pixel intensity levels for pixel *i*, pixel *j* and pixel *k*, and *I*(*x*_*n*1_, *y*_*n*1_) is the value of pixel *i*, *I*(*x*_*n*2_, *y*_*n*2_) is the value of pixel *j*, *I*(*x*_*n*3_, *y*_*n*3_) is the pixel value of *k*.

The element of the three-dimensional co-occurrence matrix is the frequency of occurrence of the gray value pair of the original image at the specified direction and distance. The number of possible neighboring pixels in the formation of 3D-GLCM is strongly influenced by the size of the original image used where the steps in building 3D-GLCM are as follows [33]:

1. Creating a frame matrix. The frame matrix shows the possible combinations of gray levels of the image and the position in the matrix
2. Forming a co-occurrence matrix. The upper left co-occurrence matrix elements will fill with the number of times the pixel combination (0, 0, 0) occurs in the image area where the combination of gray values is based on the frame matrix results
3. Transpose the co-occurrence matrix and make the matrix symmetrical. Based on the co-occurrence matrix generated in the second step, the matrix transpose operation is performed. After getting the results of the transpose matrix from the co-occurrence matrix, then form a symmetric matrix. The symmetric matrix is generated by adding the co-occurrence matrix with the transpose matrix
4. Normalization of the co-occurrence matrix. This stage is done by adding up all the elements of the symmetrical matrix. Then, the sum of all components is used as a divisor for all elements in the symmetric matrix

### 2.3. Invariant Texture Feature

A valid descriptor must possess the invariant property based on a moment where invariant is a characteristic that does not change or is unaffected by a particular transformation. Two different approaches can be used to meet the demand for invariance, namely, the basic construction of the moment invariant and the normalization of the moment [34, 35].

Equation (2) is the definition of a three-dimensional order moment.

(2) $$\begin{array}{c} \int_{0}^{1}\int_{0}^{1}\int_{0}^{1}\emptyset \left(i,j,k,g\left(p^{*}\right)\right)\omega \left(p^{*}\left(i,j,k\right)\right)d_{i}d_{j}d_{k}. \end{array}$$

The Riemann sum can approximate equations (2) and (3) where ∆_*i*_ = ∆_*j*_ = ∆_*k*_ = 1/*N* is a differential and *P* is a 3D-GLCM invariant [36].

(3) $$\begin{array}{c} \overset{N}{\underset{i=1}{\sum }}\overset{N}{\underset{j=1}{\sum }}\overset{N}{\underset{k=1}{\sum }}\emptyset \left(\frac{i}{N},\frac{j}{N},\frac{k}{N},g\left(\bar{P}\right)\right)\omega \left(\bar{P}\left(i,j,k\right)\right){∆}_{i}{∆}_{j}{∆}_{k}, \end{array}$$

(4) $$\begin{array}{c} \bar{P}=\frac{X}{\sum_{i=1}^{N}\sum_{j=1}^{N}\sum_{k=1}^{N}X\left(i,j,k\right){∆}_{i}{∆}_{j}{∆}_{k}}. \end{array}$$

There are many approaches to extracting invariant features [37–40], where the algorithm can only be used to extract variant features in the similarity transformation.

### 2.4. Feature Extraction

The power of feature extraction is the ability to measure basic patterns invisible to the human eye. In addition, the physical construction and appearance of known texture inserts make it possible to hypothesize how some texture features might behave. In this study, feature extraction was carried out using the 3D-GLCM method. The feature extraction process has two subprocesses: the formation of 3D-GLCM and the calculation of feature extraction with six statistical characteristics, namely, max probability, entropy, energy, correlation, contrast, and homogeneity.

The 3D-GLCM formation process is carried out with a distance of *d* = 1, with directions 0°, 45°, 90°, 135°, 180°, 225°, 270°, and 315°. After getting each 3D-GLCM element, it is used as a reference to calculate the value of these statistical characteristics. The calculation method of the six statistical attributes with the original 3D-GLCM is shown in Table 1.

The calculation of extracting the asymptotically invariant modified Haralick feature was based on the symmetric matrix informing the 3D-GLCM matrix without the normalization process. The analysis of statistical features is shown in Table 2, where *x* (*i*, *j*, *k*) is the *i*, *j*, *k* element in the 3D-GLCM matrix, which is not normalized, and *N* is the number of gray levels in the image. Thus, when calculating the statistical feature values (Table 2), we have

(5) $$\begin{array}{c} ∆=\frac{1}{N}, \end{array}$$

(6) $$\begin{array}{c} {∆}_{ij}=\frac{1}{N^{2}}, \end{array}$$

(7) $$\begin{array}{c} {∆}_{ijk}=\frac{1}{N^{3}}, \end{array}$$

(8) $$\begin{array}{c} {\bar{P}}_{x}\left(i\right)=\overset{N}{\underset{j=1}{\sum }}\overset{N}{\underset{k=1}{\sum }}P\left(i,j,k\right){∆}_{jk};{\bar{\mu }}_{x}=\overset{N}{\underset{i=1}{\sum }}\frac{i}{N}{\bar{P}}_{x}\left(i\right)∆, \end{array}$$

(9) $$\begin{array}{c} {\bar{P}}_{y}\left(j\right)=\overset{N}{\underset{i=1}{\sum }}\overset{N}{\underset{k=1}{\sum }}P\left(i,j,k\right){∆}_{ik};{\bar{\mu }}_{y}=\overset{N}{\underset{j=1}{\sum }}\frac{j}{N}{\bar{P}}_{y}\left(j\right)∆, \end{array}$$

(10) $$\begin{array}{c} {\bar{P}}_{z}\left(k\right)=\overset{N}{\underset{i=1}{\sum }}\overset{N}{\underset{j=1}{\sum }}P\left(i,j,k\right){∆}_{ij};{\bar{\mu }}_{z}=\overset{N}{\underset{k=1}{\sum }}\frac{k}{N}{\bar{P}}_{z}\left(k\right)∆. \end{array}$$

### 2.5. Accuracy Assessment

The performance of learning algorithms is generally measured in terms of prediction error, which gives better predictability than an independent test set. Therefore, accurate assessment of test errors is critical because it provides a reliable model selection guide and evaluation of learning methods or models [27].

Furthermore, in analyzing the effectiveness of different features, accuracy features are used to evaluate the identification results quantitatively.

In evaluating the performance of the method in this study, the reference confusion matrix is used. The confusion matrix represents the predictions and actual conditions of the data generated by the algorithm/method. The confusion matrix provides information on comparing the system's identification results with the actual identification results. The confusion matrix can measure performance in binary identification problems and multiclass identification problems [41].

Evaluation of multiclass identification ability is carried out for each class, such as evaluation for binary identification. The use of the terms “positive” and “negative” is based on predictive identification, while the terms “true” and “false” are based on whether the prediction follows the observations. Performance metrics used in the measurement include accuracy, precision, and recall. Accuracy describes how accurately the model can classify correctly, and the accuracy value can be obtained by equation (11). Precision describes the level of accuracy between the requested data and the prediction results given by the model, and this value can be obtained by equation (12). At the same time, recall describes the success of the model in rediscovering information. The recall value can be obtained by equation (13).

(11) $$\begin{array}{c} {Accuracy}_{i}=\frac{{TP}_{i}+{TN}_{i}}{{TP}_{i}+{TN}_{i}+{FP}_{i}+{FN}_{i}}, \end{array}$$

(12) $$\begin{array}{c} {Precision}_{i}=\frac{{TP}_{i}}{{TP}_{i}+{FP}_{i}}, \end{array}$$

(13) $$\begin{array}{c} {Recall}_{i}=\frac{{TP}_{i}}{{TP}_{i}+{FN}_{i}}. \end{array}$$

The given *TP*_*i*_ indicates the number of correctly identified images with the *i*th category. *FP*_*i*_ shows the number of incorrectly identified images in the *i*th category. *TN*_*i*_ shows the number of incorrectly identified images correctly. At the same time, *FN*_*i*_ is the number of correct images identified as wrong images [42, 43].

### 2.6. Image Dataset

The data in the experiment consisted of the dataset I, dataset II, brain tumor MRI dataset, and X-ray pneumonia dataset. Dataset I consists of 100 images of the right iris. Dataset II consists of 100 images of the left iris. The source of dataset I and dataset II is from the iris of an outpatient Internal Medicine Polyclinic, Airlangga University Hospital, Surabaya. The inclusion criteria of research subjects from datasets I and II were male or female. In addition, the patient did not have cataracts, and the iris was never injured or injured; the iris was not photographed after eye surgery. There are four groups in datasets I and II, namely, iris images of DM patients, HC images of patients with irises, iris images of patients with DM and HC, and iris images of normal patients. The image in the dataset is a color image (RGB) with 320 × 240pixels, and the image is saved with a .jpg file extension.

There are 215 MRI images for the MRI dataset, consisting of 140 MRI images with brain tumors and 75 MRI images with normal conditions. In addition, there are 95 X-ray images in the X-ray dataset, composed of 45 X-ray images with pneumonia and 50 X-ray images with nonpneumonia. The source of the brain tumor MRI dataset and the X-ray pneumonia dataset is from the dataset at http://kaggle.com.

## 3. Result

### 3.1. Cross-Validation

Cross-validation is a statistical method used to evaluate the performance of a model or algorithm, where the data is separated into two subsets, namely, learning process data and validation or test data. The model or algorithm is trained by the learning subset and validated by the validation subset.

**Figure 2:**
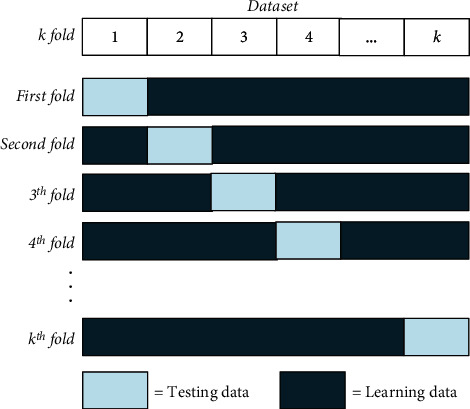
Illustration of data iteration with *k*-fold cross-validation.

In *k*-fold cross-validation, the dataset is first partitioned into *k* subsets or segments or folds of the same (or almost the identical) size, *k*-folds, namely, *D*_1_, *D*_2_, ⋯, *D*_*k*_. Then, the dataset is divided into learning data and test data. The learning and testing process was repeated *k* times [44]. The illustration of *k*-fold cross-validation data iteration is shown in Figure 2.

The workings of *k*-fold cross-validation are as follows:

1. The total dataset is divided into *k* parts
2. The 1st fold is when the 1st part becomes test data, and the rest becomes training data. Then calculate the accuracy based on that portion of the data
3. The 2nd fold is when the 2nd part becomes test data, and the rest becomes learning data. Then calculate the accuracy based on that portion of the data
4. And so on until it reaches the *k*-fold. Then, finally, calculate the average accuracy of the *k* accuracy pieces above. This average accuracy becomes the absolute accuracy

The four datasets used in this study are divided into two parts. The first data is used for the learning process by 80% of the dataset, and the second data is used for testing, which is 20% of the dataset. The testing process uses the concept of 5-fold validation. For example, in datasets I and II, the test is carried out to identify the iris image with four classes, namely, diabetes mellitus class, high cholesterol class, high cholesterol and diabetes mellitus class, and normal class. Furthermore, this study uses multiple categories to calculate the method's performance using equations (5)–(7).

The converted image to grayscale is stored in 8 bits format for each image data, allowing as many as 256 intensities. In the testing process, the image data has been tested with a smaller quantization so that the gray-level varies. Therefore, the gray-level value is converted for each dataset into 7 bits with 128 levels of gray, 6 bits with 64 levels of gray, 5 bits with 32 levels of gray, and 4 bits with 16 levels of gray.

The training process is done by finding the average value of the training data for the parameters of max probability, entropy, energy, correlation, contrast, and homogeneity. The value obtained from the training process is used to reference the testing process. This study carried out the testing process by looking for similarity values based on the Euclidean distance method. Euclidean distance is calculated using equation (14) [25].

(14) $$\begin{array}{c} d_{ij}=\sqrt[{2}]{\overset{n}{\underset{k=1}{\sum }}{\left(x_{ik}-x_{jk}\right)}^{2}.} \end{array}$$

**Figure 3:**
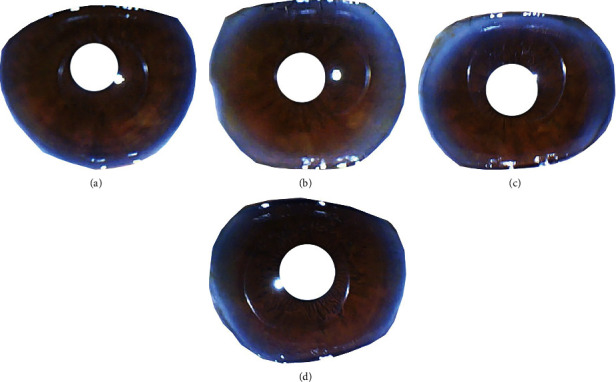
Iris image: (a) patients with DM; (b) patients with HC; (c) patients with DM and HC; (d) normal patient.

Figure 3 is an example of an iris image of a patient suffering from DM, a patient suffering from HC, a patient suffering from DM and HC, and a patient with normal DM and cholesterol levels. The image is taken from dataset I. The results of testing on the dataset I are shown in Table 3.

The results of the tests carried out on dataset II are shown in Table 4. The tests were carried out by comparing two methods, namely, the original 3D-GLCM feature extraction method with invariant 3D-GLCM feature extraction. In addition to comparing the two approaches, this test compares the differences in the gray level used in the input image.

From the testing of the dataset I and dataset II, it shows that by using the proposed 3D-GLCM modification method, namely, 3D-GLCM invariant, it gives the result that in dataset I, the accuracy has an average increase of 5.75%, the precision has an average increase of 42.24%, and the recall has an average increase of 29.01%. On the other hand, while dataset II shows an average increase in accuracy of 11.14%, precision has an average increase of 10.49%, and recall has an average increase of 15.90%.

**Figure 4:**
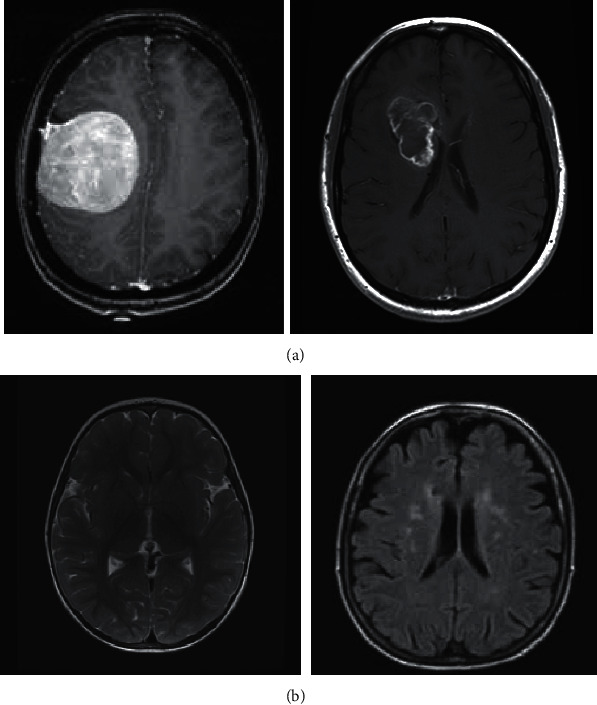
Image of MRI-brain tumor: (a) image of brain tumor patient; (b) nonbrain tumor images.

**Figure 5:**
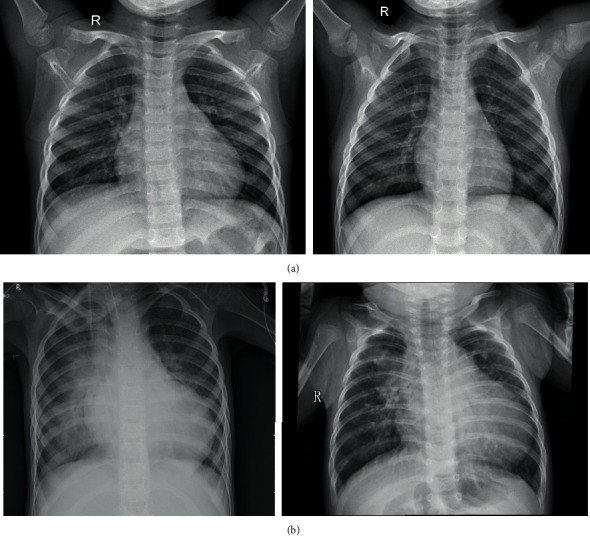
Pneumonia X-ray image: (a) image of pneumonia sufferers; (b) nonpneumonia images.

The test was also carried out on MRI-brain tumor images; an example image is shown in Figure 4 and an example of X-ray pneumonia image is shown in Figure 5.

Tables 5 and 6 are the results of tests on the MRI brain tumor dataset and the X-ray pneumonia dataset. Tests carried out on both methods also show an increase in accuracy, precision, and recall when using the invariant 3D-GLCM method.

## 4. Discussion

We have presented a modification of the Haralick texture feature, making it asymptotic invariant regarding the number of gray levels in quantization by viewing 3D-GLCM as a discrete approximation of the probability function on gray-level pairs in the image. In addition, we have shown how original 3D-GLCM feature extraction and 3D-GLCM feature extraction are invariant with increasing gray levels. We have demonstrated the benefits of the proposed asymptotically modified features by training to separate the four groups, namely, DM, CH, DM, and CH, as well as normal conditions in the I and II datasets, and to separate the two groups of brain tumor MRI images in dataset III and X-ray pneumonia images in dataset IV only based on texture features.

A classifier based on invariant features performs better than the original feature in all data tests conducted in this study. Furthermore, our proposed modification allows texture features to be reproduced regardless of quantization since the same texture features will give similar values independent of quantization.

Image requantization using fewer gray levels can produce similar images due to the limited dynamic range and repetitive texture features. The number of gray levels in quantization is essential for texture analysis because the number of gray levels determines the size of the 3D-GLCM matrix formed. Too many levels of gray can produce a sparse matrix, and too few levels of gray can have too dense of the matrix. In the statistical model built based on the number of varying levels of gray, this dramatically affects the value of the extraction feature for the max probability, entropy, energy, correlation, contrast, and homogeneity features.

Research by Garpebring et al. highlights the importance of selecting the optimal number of gray levels for texture feature calculations by showing that some GLCM-based features can achieve minimum misclassification by changing the size of the GLCM [45]. Scenarios to classify texture features were calculated with different quantization, with gray levels 16, 32, 64, 128, and 256. In this scenario, as in his research [25, 46], the quantization level is chosen to increase the features optimally in each image used. The calculation of accuracy, precision, and recall shows the gray-level variations used in all texture features. At coarse quantization where the image with a gray trim level will reduce identification accuracy, and at more significant quantization, it can increase accuracy and separability between groups. For all datasets that have been tested, the best gray level is used at 256 gray levels.

We chose to study the behavior of invariant features between and 256 gray-level quantization. Too little gray level will result in a rough Riemann approximation to equation (3). Choosing too many gray-level quantizations can result in 3D-GLCM scattering. The invariant feature ignores the same symptoms as the original feature, namely, oversensitivity to noise. Small image areas will result in sparse 3D-GLCM, underlying the failure to represent texture information properly.

In this study, we choose a global min-max limit when quantizing images on each dataset to minimize variations in feature values due to image noise or other structures in the area of interest. In addition, the global boundary approach requires that the image intensity is proportional to all images in the dataset. In this case, it requires that the image be obtained with the same hardware and imaging settings. The invariant feature can analyze images with different numbers of pixels or sizes, even to varying amounts of noise. Whereby optimizing the quantization level of each image, sambal still gets comparable texture features.

Compared to our previous research [25], the 3D-GLCM invariant modification significantly increased accuracy, precision, and recall. The test results are shown in Tables 3 and 4.

There are shortcomings in this study that could have an impact on feature repetition. The first drawback is the limited iris image dataset. The iris dataset uses the right eye and the left eye in the same patient. This limitation occurred because of the time that did not allow for primary data collection during the COVID-19 pandemic in Surabaya, Indonesia.

A larger image dataset size of a unique patient can result in better performance. The second weakness is the presence of noise in the iris image in the form of eyelashes, and the closing of the iris by the eyelids causes missing information.

Image noise will affect the value of texture features [47], but the quantization smoothing effect of the gray level can reduce the impact on the resulting features. Some features are more sensitive to noise, and more aggressive gray-level quantization for feature invariant modification can reduce the effect of noise while retaining the possibility to compare feature values with values from images analyzed at different quantization levels. This approach requires further research.

## 5. Conclusions

This paper proposes a volumetric feature extraction technique, which considers the significant differences between pixels derived from a GLCM-based feature extraction method, called 3D-GLCM. By interpreting 3D-GLCM as a discretized probability density function, it is possible to construct a set of Haralick texture features. A collection of Haralick features is modified asymptotically invariant to image quantization.

In 3D-GLCM feature extraction, the invariant retains its original interpretation. We demonstrate that invariant 3D-GLCM feature extraction can be used in different identification settings, with results superior to the original 3D-GLCM feature extraction definition. This indicates that invariant Haralick texture features can be reproduced even when different gray-level quantization is used.

## Figures and Tables

**Table 1:** Texture features calculated from 3D-GLCM.

Feature	3D-GLCM expression
Max probability	max(*p*_*ijk*_)
Entropy	$-\overset{Q}{\underset{i=1}{\sum }}\overset{Q}{\underset{j=1}{\sum }}\overset{Q}{\underset{k=1}{\sum }}p_{ijk}\left(\log_{2}p_{ijk}\right)$
Energy	$\overset{Q}{\underset{i=1}{\sum }}\overset{Q}{\underset{j=1}{\sum }}\overset{Q}{\underset{k=1}{\sum }}p_{ijk}^{2}$
Correlation	$\overset{Q}{\underset{i=1}{\sum }}\overset{Q}{\underset{j=1}{\sum }}\overset{Q}{\underset{k=1}{\sum }}p_{ijk}\frac{\left(i-\mu_{x}\right)\left(j-\mu_{y}\right)\left(k-\mu_{z}\right)}{\sigma_{x}\sigma_{y}\sigma_{z}}$
Contrast	$\overset{Q}{\underset{i=1}{\sum }}\overset{Q}{\underset{j=1}{\sum }}\overset{Q}{\underset{k=1}{\sum }}p_{ijk}\left[{\left(i-j\right)}^{2}+{\left(i-k\right)}^{2}+{\left(j-k\right)}^{2}\right]$
Homogeneity	$\overset{Q}{\underset{i=1}{\sum }}\overset{Q}{\underset{j=1}{\sum }}\overset{Q}{\underset{k=1}{\sum }}\frac{p_{ijk}}{\left[{\left(i-j\right)}^{2}+{\left(i-k\right)}^{2}+{\left(j-k\right)}^{2}\right]}$

**Table 2:** Texture features calculated from 3D-GLCM invariant.

Feature	3D-GLCM invariant expression
Max probability	$\max\left({\bar{p}}_{ijk}\right)$
Entropy	$-\overset{N}{\underset{i=1}{\sum }}\overset{N}{\underset{j=1}{\sum }}\overset{N}{\underset{k=1}{\sum }}{\bar{p}}_{ijk}\left(\log_{2}{\bar{p}}_{ijk}\right){∆}_{ijk}$
Energy	$\overset{N}{\underset{i=1}{\sum }}\overset{N}{\underset{j=1}{\sum }}\overset{N}{\underset{k=1}{\sum }}{\bar{p}}_{ijk}^{2}{∆}_{ijk}$
Correlation	$\overset{N}{\underset{i=1}{\sum }}\overset{N}{\underset{j=1}{\sum }}\overset{N}{\underset{k=1}{\sum }}{\bar{p}}_{ijk}\frac{\left(i/N-{\bar{\mu }}_{x}\right)\left(j/N-{\bar{\mu }}_{y}\right)\left(k/N-{\bar{\mu }}_{z}\right)}{{\bar{\sigma }}_{x}{\bar{\sigma }}_{y}{\bar{\sigma }}_{z}}$
Contrast	$\overset{N}{\underset{i=1}{\sum }}\overset{N}{\underset{j=1}{\sum }}\overset{N}{\underset{k=1}{\sum }}{\bar{p}}_{ijk}\left[{\left(\frac{i}{N}-\frac{j}{N}\right)}^{2}+{\left(\frac{i}{N}-\frac{k}{N}\right)}^{2}+{\left(\frac{j}{N}-\frac{k}{N}\right)}^{2}\right]{∆}_{ijk}$
Homogeneity	$\overset{N}{\underset{i=1}{\sum }}\overset{N}{\underset{j=1}{\sum }}\overset{N}{\underset{k=1}{\sum }}\frac{{\bar{p}}_{ijk}{∆}_{ijk}}{1+\left[{\left(i/N-j/N\right)}^{2}+{\left(i/N-k/N\right)}^{2}+{\left(j/N-k/N\right)}^{2}\right]}$

**Table 3:** Test results on the dataset I.

Method	3D-GLCM (original)			3D-GLCM invariant		
Level Grayscale	Acc	Prec	Recall	Acc	Prec	Recall
16	69.79	33.80	39.58	73.44	50.94	47.92
32	69.27	35.94	45.83	71.88	53.08	52.08
64	71.35	34.11	37.50	75.00	51.04	54.17
128	72.92	40.31	45.83	82.81	65.10	75.00
256	96.88	95.31	93.75	97.92	96.88	95.83

Acc: accuracy; Prec: precision.

**Table 4:** Test results on the dataset II.

Method	3D-GLCM (original)			3D-GLCM invariant		
Level Grayscale	Acc	Prec	Recall	Acc	Prec	Recall
16	71.88	41.67	43.75	73.96	45.01	47.92
32	66.25	69.44	57.50	78.75	80.71	63.5
64	71.25	76.52	62.50	71.88	82.19	75.58
128	73.75	79.89	65.00	87.50	90.18	85.00
256	83.75	83.13	85.00	95.83	89.69	91.67

Acc: accuracy; Prec: precision.

**Table 5:** Test results on the brain tumor MRI dataset.

Method	3D-GLCM (original)			3D-GLCM invariant		
Level Grayscale	Acc	Prec	Recall	Acc	Prec	Recall
16	60.83	61.22	65.00	90.00	87.67	95.00
32	60.83	61.40	65.00	92.50	87.91	100.00
64	70.00	69.19	70.00	92.50	89.77	96.67
128	81.25	79.32	85.00	96.67	95.39	98.33
256	87.50	87.47	88.33	97.50	97.06	98.33

Acc: accuracy; Prec: precision.

**Table 6:** Test results on the X-ray pneumonia dataset.

Method	3D-GLCM (original)			3D-GLCM invariant		
Level Grayscale	Acc	Prec	Recall	Acc	Prec	Recall
16	58.75	58.84	76.00	86.25	94.38	77.50
32	66.25	67.25	65.00	72.50	77.16	67.50
64	77.50	76.36	80.00	87.50	88.79	87.50
128	83.75	84.83	82.50	96.25	93.18	100.00
256	86.25	85.63	87.50	96.25	93.18	100.00

## Data Availability

The datasets used in the research can be publicly accessed through the bellow links: **(**1) dataset I (dataset iris): https://www.kaggle.com/rincikembanghapsari/dataset-i-iris; (2) dataset II (dataset iris): https://www.kaggle.com/rincikembanghapsari/dataset-ii-iris; (3) MRI brain tumor: https://www.kaggle.com/navoneel/brain-mri-images-for-brain-tumor-detection; (4) X-ray pneumonia: https://www.kaggle.com/paultimothymooney/chest-xray-pneumonia
